# Draft Whole-Genome Sequence of a Novel Chryseobacterium viscerum Strain Isolated from Fresh Water at Dripping Springs, New Mexico

**DOI:** 10.1128/MRA.01155-19

**Published:** 2019-10-10

**Authors:** John A. Kyndt, Tyler C. Moore

**Affiliations:** a College of Science and Technology, Bellevue University, Bellevue, Nebraska, USA; Georgia Institute of Technology

## Abstract

We sequenced the genome of a bacterial species recently isolated from fresh water at Dripping Springs, NM, and identified it as Chryseobacterium viscerum. This species had previously been isolated only from dead or diseased fish. This report shows that C. viscerum can be found in nature as a free-living species not associated with diseased fish.

## ANNOUNCEMENT

The genus Chryseobacterium belongs to the family *Flavobacteriaceae*, and several of its members have been reported to be pathogens (1–3). In addition to often being associated with diseased and apparently healthy fish, several Chryseobacterium spp. have been described to exhibit plant growth promotion and act as a biocontrol against plant pathogens (2, 4–7). The species Chryseobacterium viscerum has been described as isolated only from diseased fish, particularly rainbow trout (2, 3). Only one report thus far has reported this species in North America, in a study of flavobacteriosis-associated species in Michigan (8). These data suggest that C. viscerum is a potential fish pathogen; however, there have been no studies on the virulence of this species or the pathogen-host interactions, or even whether or not C. viscerum can be found as a free-living species.

We recently performed a metagenome analysis of water samples isolated from Dripping Springs, NM, and identified a wide diversity of new species (J. A. Kyndt, submitted for publication). Upon plating the isolated spring samples on nutrient agar and incubating at 25°C, we observed several distinctive orange-colored colonies. The orange isolate was purified by repetitive streaking on nutrient agar medium (Carolina Biological Supply), and genomic DNA was isolated using the GeneJET DNA purification kit (Thermo Scientific). Utilizing a Qubit fluorometer and NanoDrop spectrophotometer, we determined the quality and quantity of DNA, showing a 260/280 absorbance ratio of 1.83. A partial 16S rRNA gene was PCR amplified using the fD1 and rP1 primers described by Weisburg et al. (9). A BLAST (NCBI) comparison of the partial 16S rRNA sequences showed the top 10 hits to be various *Chryseobacterium* species with 97 to 98% identity. To further characterize the species, we sequenced the whole genome using an Illumina MiniSeq platform, using 500 μl of a 1.8 pM library prepared with the Nextera DNA Flex library prep kit. Paired-end (2 × 150 bp) sequencing generated 1,354,974 reads and 109 Mbp. Quality control of the reads was performed using FastQC within BaseSpace version 1.0.0 (Illumina), using a k-mer size of 5 and contamination filtering. We then assembled the genome *de novo* using SPAdes version 3.10.0 (10) through PATRIC, using default parameters (11). This assembly showed our strain to be 5,153,060 bp long and yielded 35 contigs (≥500 bp), with the largest being 760,151 bp, and the *N*_50_ value was 339,377 bp. The GC content was 36.2%. We then annotated the genome sequence using the RAST tool kit (RAST*tk*) (12) within PATRIC, using default parameters (11), which identified 4,793 coding sequences and 84 tRNAs.

A JSpecies comparison (13) of the average nucleotide identity (ANI) between this genome and other Chryseobacterium genomes gave the following percentages: 97.9%, *C. viscerum* 687B-08; 85.2%, *C. gleum*; 85.0%, *C. culicis* DSM 23031; 82.0%, *C. arthrosphaerae* CC-VM-7; 81.5%, *C. contaminans* DSM 27621; and 80.9%, *C. indologenes* DSM 16777. Thus, our strain clearly belongs to C. viscerum, and we designated it Chryseobacterium viscerum strain DPS (Dripping Springs). All other chryseobacteria that have been sequenced have less than 80% ANI and are clearly below the proposed 95% cutoff for genome definition of a species (13).

To our knowledge, this is the first report of a free-living C. viscerum strain not isolated from diseased fish. The species was isolated from a water source where no fish species were found at the time of sampling, and moreover, the size of the stream in combination with the arid and mountainous topography is not likely to accommodate any fish species (https://www.blm.gov/visit/dripping-springs-natural-area and https://www.desertusa.com/desert-new-mexico/ruins-dripping-springs.html). This report suggests that C. viscerum is a free-living microbe that, based on its previous isolations from dead or diseased fish, could be capable of opportunistically infecting teleost fish.

### Data availability.

This whole-genome shotgun project has been deposited at DDBJ/ENA/GenBank under the accession number VTPV00000000. The version described in this paper is version VTPV01000000. The raw sequencing reads have been submitted to the SRA with accession number SRR10056317.
